# Exact statistical solution for the hopping transport of trapped charge via finite Markov jump processes

**DOI:** 10.1038/s41598-021-89280-7

**Published:** 2021-05-13

**Authors:** Andrey A. Pil’nik, Andrey A. Chernov, Damir R. Islamov

**Affiliations:** 1 Rzhanov Institute of Semiconductor Physics, Siberian Branch of the Russian Academy of Sciences, Novosibirsk, Russian Federation 630090; 2 Novosibirsk State University, Novosibirsk, Russian Federation 630090

**Keywords:** Electronic and spintronic devices, Surfaces, interfaces and thin films, Electronic devices

## Abstract

In this study, we developed a discrete theory of the charge transport in thin dielectric films by trapped electrons or holes, that is applicable both for the case of countable and a large number of traps. It was shown that Shockley–Read–Hall-like transport equations, which describe the 1D transport through dielectric layers, might incorrectly describe the charge flow through ultra-thin layers with a countable number of traps, taking into account the injection from and extraction to electrodes (contacts). A comparison with other theoretical models shows a good agreement. The developed model can be applied to one-, two- and three-dimensional systems. The model, formulated in a system of linear algebraic equations, can be implemented in the computational code using different optimized libraries. We demonstrated that analytical solutions can be found for stationary cases for any trap distribution and for the dynamics of system evolution for special cases. These solutions can be used to test the code and for studying the charge transport properties of thin dielectric films.

## Introduction

New dielectrics, like high-$\kappa$ ${\hbox {HfO}_2}$, ${\hbox {Ta}_{2}\hbox {O}_{5}}$, ${\hbox {ZrO}_{2}}$^1–5^ and low-$\kappa$ ones^6–9^, are extremely perspective materials for using them in modern and future electronic devices, including memory and digital ones. That is why the knowledge about the charge transport processes and mechanisms in dielectrics is critical for modern microelectronics. High-$\kappa$ dielectrics are promising materials for resistive random access memory (RRAM), ferroelectric random access memory (FRAM) and memristor devices, while low-$\kappa$ dielectrics are the insulating dielectrics that separate wire interconnects and transistors from each other in high-speed integrated circuits^6^. High-$\kappa$-based MOSFETs, FRAM and flash memories, and low-$\kappa$ interconnect separators need dielectrics with low leakage currents, while RRAM devices exhibit a better performance when the used dielectric layers are mostly nonstoichiometric, i.e. include a lot of defects and traps^10–12^. That is why producing high-quality electronic devices requires a strict control of deposited dielectric film quality to monitor leakage currents.

The charge currents in metal-insulator-semiconductor (MIS) and metal-insulator-metal structures (MIM) might be limited by a contact metal-insulator or insulator-semiconductor (contact-limited currents) or by the traps in the insulator bulk (bulk- or trap-limited currents). Contact-limited current models describe the electron and hole emission from semiconductor or metal electrodes to traps (localized states in the bandgap) or to the conduction band in dielectrics. The quantum field emission, first proposed by R. Fowler and L. Nordheim, describes the charge-carriers tunnelling through a triangle energy barrier to the dielectric conduction band and does not depend on temperature^13^. At high temperatures, a significant contribution to the transport might be given by the thermally assisted tunnelling^14,15^ and field-enhanced thermionic emission of hot carriers (Schottky effect)^16^. The direct tunnelling from one electrode to another through a trapeze barrier takes place in the structures based on ultra-thin ($<5$ nm) dielectric films^17^.

Bulk-limited charge transport models can be divided into two large subgroups: isolated trap ionisation (Frenkel model^18,19^, multiphonon single trap ionization proposed by Makram-Ebeid and Lannoo^20^) and hops between neighbouring traps (thermally assisted traps between overlapped Coulomb centres^21,22^, phonon-assisted tunnelling of electrons (holes) between neighbouring traps^23^).

In essence, all these models give the probabilities of a hop between two traps and between a trap and an electrode. Usually, a set of approaches was used to describe the charge transport between traps in dielectric layers using these probabilities. One on them is the time-dependent direct or Monte-Carlo simulations with hops between traps, and it requires carrying out many simulations to obtain sufficient statistics for data averaging in order to identify general patterns. Another approach is discrete Shockley–Read–Hall-like (SRH) 1D transport equations with the boundary conditions^23,24^:

1 $$\begin{aligned} \begin{aligned} \frac{\hbox {d}{\bar{n}}_{\alpha }}{\hbox {d}t}&= {\bar{n}}_{\alpha -1}(1 - {\bar{n}}_{\alpha }){H}_{\alpha -1,\alpha } - {\bar{n}}_{\alpha }(1 - {\bar{n}}_{\alpha -1}){H}_{\alpha -1, \alpha } + {\bar{n}}_{\alpha +1}(1 - {\bar{n}}_{\alpha }){H}_{\alpha +1,\alpha } - {\bar{n}}_{\alpha }(1 - {\bar{n}}_{\alpha +1}){H}_{\alpha , \alpha +1} + G_\alpha - R_\alpha ;\\ \frac{\hbox {d}{\bar{n}}_{1}}{\hbox {d}t}&= (1 - {\bar{n}}_{1}){I}_1 - {\bar{n}}_{1}{E}_1 + {\bar{n}}_{2}(1 - {\bar{n}}_{1}){H}_{2,1} - {\bar{n}}_{1}(1 - {\bar{n}}_{2}){H}_{1, 2} + G_1 - R_1;\\ \frac{\hbox {d}{\bar{n}}_{N}}{\hbox {d}t}&= (1 - {\bar{n}}_{N}){I}_{N} - {\bar{n}}_{N}{E}_N + {\bar{n}}_{N-1}(1 - {\bar{n}}_{N}){H}_{N-1,N} - {\bar{n}}_{N}(1 - {\bar{n}}_{N-1}){H}_{N, N-1} + G_N - R_N, \end{aligned} \end{aligned}$$

where ${\bar{n}}_{\alpha }$ is the average filling of the $\alpha$th trap, *t* is time, ${H}_{\alpha , \beta }$ is the probability rate of a hop from the $\alpha$th trap to the $\beta$th one, $G_\alpha$ is the trapped carrier generation rate (due to free carriers localization) and $R_\alpha$ is the trapped carrier recombination rate (due to the trap ionization to a conduction/valence band, recombination with free carriers with an opposite sign) and *N* is the trap number. In case of low free charge carriers density $n_{\mathrm{t}}\gg n$ (here $n_{\mathrm{t}}$ is the trapped carrier density, *n* if the free carriers density), the terms $G_\alpha$ and $R_\alpha$ may be omitted due to their smallness. Recently, discrete equation (1) has been extended to a continuous model^25^:

2 $$\begin{aligned} \frac{\partial {\bar{n}}}{\partial t} = - a \frac{\partial }{\partial x} \{ [{H}^+ - {H}^-] {\bar{n}}(1 - {\bar{n}})\} +\dfrac{a^2}{2} \frac{\partial }{\partial x} \left\{ [{H}^+ + {H}^-] \frac{\partial {\bar{n}}}{\partial x}\right\} , \end{aligned}$$

where *a* is the distance between traps, *x* is the coordinate, ${H}^\pm = {H}(\pm F)$ are the hop rates between traps along and against electric field *F*. This approach is approximate, but it works fine for a relatively large number of traps in a dielectric medium. Modern electronic devices based on thin films are produced with the feature size of several nanometers. In this case, the number of traps in dielectric bulk *N* is countable, and the filling averaging over them might give quite large deviations due to the $\sqrt{N}/N=1/\sqrt{N}$ law.

All these methods are approximate due to different reasons. Monte-Carlo simulations are approximate by the virtue of them being a numerical calculation method. Both discrete and continuous SRH strongly rely on the fact that fillings of neighboring traps are statistically independent. In fact, they are obviously not independent. That is why the SRH approach is an approximate one. Furthermore, time-dependent SRH equations should be integrated numerically, and it adds an error to the result. The approximate nature of all these methods require using a validation by, for example, some exact analytical solutions as a reference. To obtain such solutions a new model needs to be developed. The aim of the present work is the development of an exact universal model of trapped charge transport via a hopping between traps in strong electric fields applicable both for the case of countable and a large number of traps.

## Results

### Markov-jump-like (MJ) model formulation

Let us consider the volume of a dielectric containing *N* traps. From the classical standpoint (considering electrons to be strongly localized inside traps) there is a finite set of $2^N$ different variants of charge localization inside traps (states): ${\varvec{n}}^1, {\varvec{n}}^2, \dots , {\varvec{n}}^{2^N}$. Each state ${\varvec{n}}^\alpha$ has a certain probability of transition to state ${\varvec{n}}^\beta$ during a short period of time $\delta t$, which we will indicate as $P_{\alpha , \beta }$. Obviously, the probability $P_{\alpha , \alpha }$ of not changing state ${\varvec{n}}^\alpha$ can be obtained from the probabilities of all transitions from ${\varvec{n}}^\alpha$: $P_{\alpha , \alpha } = 1 - \sum _{\beta \ne \alpha } P_{\alpha , \beta }$. Thus, the transition probability matrix ${\hat{\mathbf{P }}}$ sized $2^N \times 2^N$ can be compiled.

At each moment in time, the system can be characterized by the column vector ${\varvec{p}} = [p_1;\, p_2;\, \dots ;\, p_{2^N}]$ of probabilities, where $p_\alpha$ is the probability of finding the system in state ${\varvec{n}}^\alpha$. The average state evolution of the system during one time step $\delta t$ can now be described by the expression ${\varvec{p}}(t+\delta t) = {\hat{\mathbf{P }}^{\mathrm{T}}} {\varvec{p}}(t)$, which can be transformed to the form ${\varvec{p}}(t + \delta t) - {\varvec{p}}(t) = ({\hat{\mathbf{P }}}^{\mathrm{T}} - {\hat{\mathbf{I }}}){\varvec{p}}(t)$, where ${\hat{\mathbf{I }}}$ is the identity matrix. The differential form of describing the evolution of ${\varvec{p}}$ can be achieved by considering the limit $\delta t \rightarrow 0$:

3 $$\begin{aligned} \frac{\hbox {d}{\varvec{p}}}{\hbox {d}t} = {\hat{\mathbf{Q }}}{\varvec{p}}, \quad \text {where} \quad {\hat{\mathbf{Q }}} = \lim \limits _{\delta t \rightarrow 0} \dfrac{{\hat{\mathbf{P }}}^{\mathrm{T}} - {\hat{\mathbf{I }}}}{\delta t}. \end{aligned}$$

This equation is known as a Kolmogorov backward equation (KBE). The element $Q_{\alpha , \beta }$ of matrix ${\hat{\mathbf{Q }}}$ can be treated as the rate of transition from state ${\varvec{n}}^\beta$ to state ${\varvec{n}}^\alpha$ (so, $Q_{\alpha , \beta } \delta t$ is the probability of such a transition in short time $\delta t$). Diagonal elements can again be found from all of the rates of transition from the state ${\varvec{n}}^\alpha$: $Q_{\alpha , \alpha } = - \sum _{\beta \ne \alpha } Q_{\beta , \alpha }$. The resulting differential equation (3) can be solved analytically:

4 $$\begin{aligned} {\varvec{p}}(t) = \exp ({\hat{\mathbf{Q }}}t) {\varvec{p}}(0). \end{aligned}$$

If we represent the state ${\varvec{n}}^\gamma$ as a column vector ${\varvec{n}}^\gamma = [n_1^\gamma ;\, n_2^\gamma ;\, ...;\, n_N^\gamma ]$, where $n_\alpha ^\gamma$ is the filling of the $\alpha$th trap in state $\gamma$ (takes the value 0 if the trap is empty and the value 1 if a charge carrier is captured in the trap), then the average ensemble filling of traps $\bar{{\varvec{n}}} = [{\bar{n}}_1;\, {\bar{n}}_2;\, ...;\, {\bar{n}}_N]$ can be expressed as an average for all states according to their individual probability values:

$$\begin{aligned} \bar{{\varvec{n}}}(t) = \sum _{\gamma =1}^{2^N} {\varvec{n}}^\gamma p_\gamma (t) = {\hat{\mathbf{N }}}{\varvec{p}}(t) = {\hat{\mathbf{N }}}\exp ({\hat{\mathbf{Q }}}t) {\varvec{p}}(0), \end{aligned}$$

where ${\hat{\mathbf{N }}}$ is the matrix of states such that $N_{\alpha ,\beta }$ is the occupancy of the $\alpha$th trap in the $\beta$th state.

Each option of filling traps ${\varvec{n}}^\gamma$ is characterized by the matrix of currents ${\hat{\mathbf{i }}}^\gamma$, where $i^\gamma _{\alpha , \beta }$ is the current between the $\alpha$th and $\beta$th traps in state $\gamma$. Obviously, the matrix is skew-symmetric: $i^\gamma _{\alpha , \beta } = - i^\gamma _{\beta , \alpha }$. So, the matrix of currents can be expressed as an average for all states according to their individual probability values:

$$\begin{aligned} {\hat{\mathbf{i }}}(t) = \sum _{\gamma =1}^{2^N } {\hat{\mathbf{i }}}^\gamma p_\gamma (t). \end{aligned}$$

The current between electrodes and traps can also be introduced in this matrix by adding the corresponding rows and columns to the matrix. Indeed, we can treat each electrode as an additional trap with the corresponding currents towards/from all of the other traps. For example, one can treat the left and right electrode as $(N + 1)$th and $(N+2)$th traps, correspondingly, so that $i^\gamma _{N+1, \beta }$ and $i^\gamma _{\beta , N+1}$ are the currents from the left electrode to the $\beta$th trap and the current from the $\beta$th trap towards the left electrode; $i^\gamma _{N + 2, \beta }$ and $i^\gamma _{\beta , N + 2}$ are similar currents for the right electrode.

### Stationary state

In many cases, the desired characteristic of a system is the stationary state achieved with time and not the dynamics of its development. In a stationary state, the column vector ${\varvec{p}}$ stops changing. Therefore, from (3) it follows that ${\hat{\mathbf{Q}}}{\varvec{p}}^{{{\text{st}}}} = 0$. Also, by virtue of its definition, the probability vector ${\varvec{p}}$ must always satisfy the additional constraint that the sum of its elements is equal to 1. This means that to find a stationary vector of probabilities, it is necessary to solve a system of $2^N + 1$ linear equations:

5 $$\begin{aligned} \begin{array}{ccccccccc} p^{\hbox{st}}_1 &{} + &{} p^{\hbox{st}}_2 &{} + &{} \dots &{} + &{} p^{\hbox{st}}_{2^N}&{} = &{} 1 \\ Q_{1,1} p^{\hbox{st}}_1 &{} + &{} Q_{1,2} p^{\hbox{st}}_2 &{} + &{} \dots &{} + &{} Q_{1,2^N} p^{\hbox{st}}_{2^N} &{} = &{} 0 \\ Q_{2,1} p^{\hbox{st}}_1 &{} + &{} Q_{2,2} p^{\hbox{st}}_2 &{} + &{} \dots &{} + &{} Q_{2,2^N} p^{\hbox{st}}_{2^N} &{} = &{} 0 \\ \vdots &{}&{}\vdots &{}&{}&{}&{}\vdots &{}&{}\vdots \\[2pt] Q_{2^N,1} p^{\hbox{st}}_1 &{} + &{} Q_{2^N,2} p^{\hbox{st}}_2 &{} + &{} \dots &{} + &{} Q_{2^N,2^N} p^{\hbox{st}}_{2^N} &{} = &{} 0. \\ \end{array} \end{aligned}$$

It can be solved as is or can be converted to a more standard form. Obviously, the system is overdetermined (the number of equations is larger than the number of the ‘unknown’). However, the conventional square non-homogeneous system can also be produced from it in a number of ways. One way is to use the result of adding the first equation to the rest $2^N$ equations:

$$\begin{aligned} \begin{array}{ccccccccc} (1 + Q_{1,1}) p^{\hbox{st}}_1 &{} + &{} (1 + Q_{1,2}) p^{\hbox{st}}_2 &{} + &{} \dots &{} + &{} (1 + Q_{1,2^N}) p^{\hbox{st}}_{2^N} &{} = &{} 1 \\ (1 + Q_{2,1}) p^{\hbox{st}}_1 &{} + &{} (1 + Q_{2,2}) p^{\hbox{st}}_2 &{} + &{} \dots &{} + &{} (1 + Q_{2,2^N}) p^{\hbox{st}}_{2^N} &{} = &{} 1 \\ \vdots &{}&{}\vdots &{}&{}&{}&{}\vdots &{}&{}\vdots \\ (1 + Q_{2^N,1}) p^{\hbox{st}}_1 &{} + &{} (1 + Q_{2^N,2}) p^{\hbox{st}}_2 &{} + &{} \dots &{} + &{} (1 + Q_{2^N,2^N}) p^{\hbox{st}}_{2^N} &{} = &{} 1. \\ \end{array} \end{aligned}$$

The system of equations can be expressed in the matrix form:

$$\begin{aligned} ({\hat{\mathbf{J }}} + {\hat{\mathbf{Q }}}){\varvec{p}}^{\hbox{st}}= {\varvec{j}}, \end{aligned}$$

where ${\hat{\mathbf{J }}}$ is the matrix of ones, ${\varvec{j}}$ is the vector of ones. Now, using the matrix notation, the stationary state characteristics can be expressed explicitly:

$$\begin{aligned} {\varvec{p}}^{\hbox{st}}= ({\hat{\mathbf{J }}} + {\hat{\mathbf{Q }}})^{-1} {\varvec{j}}, \qquad \bar{{\varvec{n}}}^{\hbox{st}}= {\hat{\mathbf{N }}}({\hat{\mathbf{Q }}} + {\hat{\mathbf{J }}})^{-1} {\varvec{j}}. \end{aligned}$$

It is important to note that, despite the mathematical elegance, this way of expressing the solution may not be the most optimal one. Depending on the numerical methods in use, it might be wise to keep the sparsity of matrices. The sparse square non-homogeneous form can be achieved, for example, by simply removing the second equation from the system (5). Indeed, as far as $\sum \nolimits _{\beta =1}^{2^N} Q_{\beta ,\alpha }=0$, one could see that the sum of the last $2^N - 1$ equations in (5) is equivalent to the second equation. So, the second equation can be discarded as linearly dependent on other equations. It should be noted that the same is true for any equation in (5) except for the first one. Now the matrix form of this system of equations is:

$$\begin{aligned} \hat{\mathbf{Q}}_1 \mathbf{p}^{\hbox{st}}= {\varvec{j}}_1, \end{aligned}$$

where ${\hat{\mathbf{Q }}}_1$ is the matrix ${\hat{\mathbf{Q }}}$ with the elements of the first row substituted with ones, ${\varvec{j}}_1$ is the vector, such that the first element is equal to 1 and the rest of the elements are equal to 0. The explicit form of the stationary characteristics in this case is:

$$\begin{aligned} {\varvec{p}}^{\hbox{st}}= {\hat{\mathbf{Q }}}_1 ^{-1} {\varvec{j}}_1, \qquad \bar{{\varvec{n}}}^{\hbox{st}}= {\hat{\mathbf{N }}}{\hat{\mathbf{Q }}}_1 ^{-1} {\varvec{j}}_1. \end{aligned}$$

It can also be seen that column vector ${\varvec{p}}^{\hbox{st}}$ can be found as the first column of matrix ${\hat{\mathbf{Q }}}_1 ^{-1}$, and column vector $\bar{{\varvec{n}}}^{\hbox{st}}$ can be found as the first column of matrix ${\hat{\mathbf{N }}}{\hat{\mathbf{Q }}}_1 ^{-1}$ (due to the associative property of matrix multiplication).

### Hop current model

The elementary acts of hopping conduction in the model are:

- hopping of one charge carrier from an filled trap to an empty one. The rate of such event will be indicated as ${H}$;
- extraction (ionization and departure) of one carrier from a filled trap towards an electrode. The rate of such event will be indicated as ${E}$;
- injection of one carrier from an electrode into an empty trap. The rate of such event will be indicated as ${I}$.

The probability of observing an elementary act during the time $\delta t$ is the product of $\delta t$ and the rate of this act. Thus, the probability of observing the event consisting of *L* elementary acts during the time $\delta t$ will be proportional to $\delta t^L$. This means that an addition to matrix ${\hat{\mathbf{P }}}$ from all events consisting of more than one elementary act does not affect the matrix ${\hat{\mathbf{Q }}}$ according to its definition. Indeed, the limit in (3) gives 0 for any addition with $L > 1$. So, considering the events taking place during the transition from one state to another, we can limit ourselves just to the events consisting of one elementary act. However, some transitions are not achievable by only one elementary act. For example, if the state ${\varvec{n}}^\alpha$ differs from the state ${\varvec{n}}^\beta$ in more than 2 places, the rate of such transition is zero ($Q_{\alpha , \beta } = 0$). The number of possible non-zero elements and their fraction in matrix ${\hat{\mathbf{Q }}}$ can be easily calculated:

6 $$\begin{aligned} N_{\mathrm {nz}} = 2^{N-2} (N^2 + 3N + 4), \qquad \rho = \dfrac{N_{\mathrm {nz}}}{2^{2 N}} = \dfrac{N^2 + 3N + 4}{2^{N + 2}}. \end{aligned}$$

**Figure 1:**
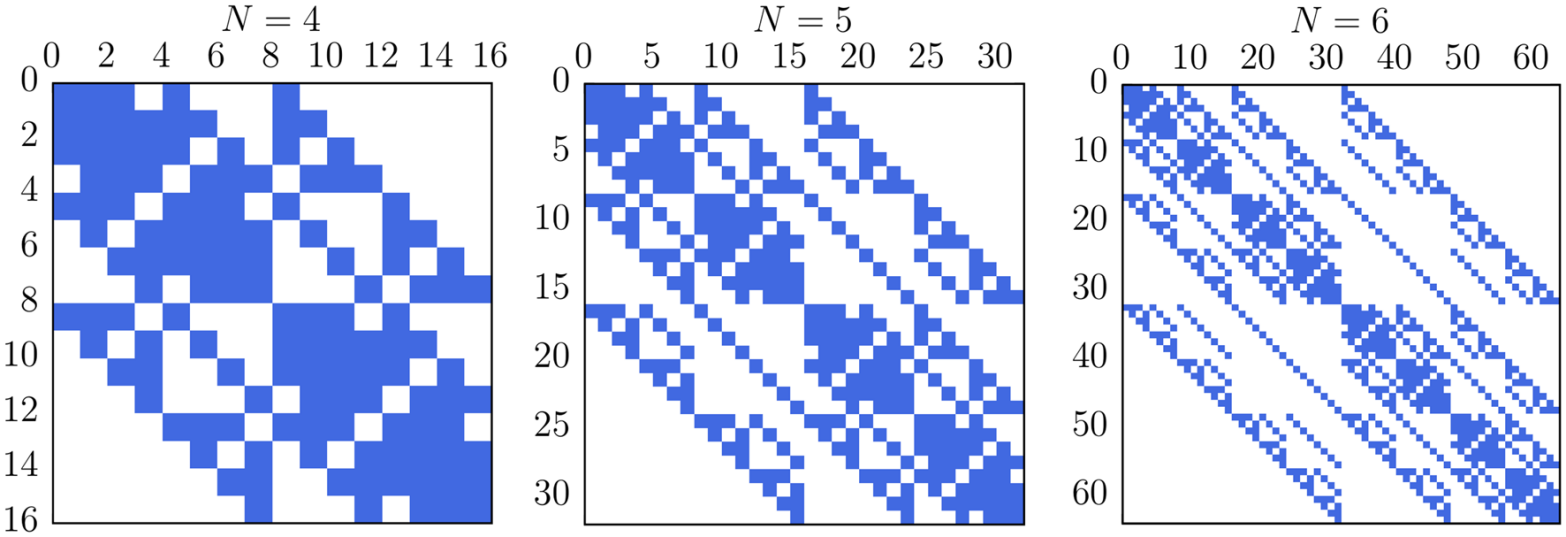
Illustration of sparsity of matrix ${\hat{\mathbf{Q }}}$ for $N=4$, 5 and 6. White corresponds to zero-valued elements (impossible transitions).

Thus, even the most general form of matrix ${\hat{\mathbf{Q }}}$ can be considered sparse (contains mostly zeros) for a reasonably large number of traps, as shown in Fig. 1.

Many of the indicated possible non-zero elements can also be treated as zeros due to the fact that the probability of charge transitions is extremely dependent on the distance and the hopping of a trapped charge carrier into a trap not adjacent to it (far jump) is unlikely, compared to a jump into the neighboring one. The same applies to the carrier injection from the electrode into an empty trap and extraction from an occupied trap towards the electrode. So, for the simplicity of computations, one can limit oneself to considering only short-range events.

## Examples

In this section we will apply the model to some simple cases of the hopping conduction to illustrate the general mathematical formulation.

### Two traps in a strong electric field

**Figure 2:**
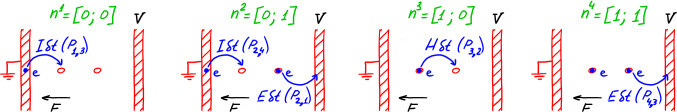
Illustration of different states ${\varvec{n}}^\alpha$ and electron transition probability values $P_{\alpha , \beta }$ for $N=2$ under the strong right-to-left electric field.

Consider a simple case of a 2-trap chain under the strong right-to-left electric field, as illustrated in Fig. 2. Under such conditions, we can neglect the probability of electrons moving to the left. In this case, only 3 short-range events can happen: injection from the left electrode into the first trap, jumping of the electron trapped in the first trap towards the second trap and extraction of the electron trapped in the second trap towards the right electrode. In this case the considered matrices are as follows:

7 $$\begin{aligned} {\hat{\mathbf{P }}}= & {} \begin{bmatrix} 1 - {I}\delta t &{} 0 &{} {I}\delta t &{} 0\\ {E}\delta t &{} 1 - {E}\delta t - {I}\delta t &{} 0 &{} {I}\delta t\\ 0 &{} {H}\delta t &{} 1 - {H}\delta t &{} 0\\ 0 &{} 0 &{} {E}\delta t &{} 1 - {E}\delta t\\ \end{bmatrix}, \quad {\hat{\mathbf{Q }}} = \lim \limits _{\delta t \rightarrow 0} \dfrac{{\hat{\mathbf{P }}}^{\mathrm{T}} - {\hat{\mathbf{I }}}}{\delta t} = \begin{bmatrix} - {I}&{E} &{} 0&{} 0\\ {} 0&{} - {E}- {I}&{H} &{} 0\\ {I} &{} 0&{} - {H}&{E} \\ 0 &{I} &{} 0&{} - {E}\\ \end{bmatrix}, \end{aligned}$$

8 $$\begin{aligned} {\hat{\mathbf{i }}}^1= & {} \begin{bmatrix} 0 &{} 0 &{} -q {I}&{} 0\\ 0 &{} 0 &{} 0 &{} 0\\ q {I}&{} 0 &{} 0 &{} 0\\ 0 &{} 0 &{} 0 &{} 0\\ \end{bmatrix}, \quad {\hat{\mathbf{i }}}^2 = \begin{bmatrix} 0 &{} 0 &{} -q {I}&{} 0\\ 0 &{} 0 &{} 0 &{} q {E}\\ q {I}&{} 0 &{} 0 &{} 0\\ 0 &{} -q {E}&{} 0 &{} 0\\ \end{bmatrix}, \quad {\hat{\mathbf{i }}}^3 = \begin{bmatrix} 0 &{} q {H}&{} 0 &{} 0\\ -q {H}&{} 0 &{} 0 &{} 0\\ 0 &{} 0 &{} 0 &{} 0\\ 0 &{} 0 &{} 0 &{} 0\\ \end{bmatrix},\quad {\hat{\mathbf{i }}}^4 = \begin{bmatrix} 0 &{} 0 &{} 0 &{} 0\\ 0 &{} 0 &{} 0 &{} q {E}\\ 0 &{} 0 &{} 0 &{} 0\\ 0 &{} -q {E}&{} 0 &{} 0\\ \end{bmatrix}, \end{aligned}$$

where *q* is the signed carrier charge (equal to the elementary charge modulo).

Solving equations (5) using (7) gives us the stationary probability values of different states:

9 $$\begin{aligned} {\varvec{p}}^{\hbox{st}}=\dfrac{1}{({E}+{H})({E}+ {I}){I}+ {E}^2{H}} [{E}^2 {H};\, {E}{I}{H};\, {E}{I}( {E}+ {I});\, {I}^2{H}]. \end{aligned}$$

The average stationary filling can be calculated using the trap filling in different states, and the found stationary probabilities of these states (9) are

$$\begin{aligned} \bar{{\varvec{n}}}^{\hbox{st}}= & {} \sum _{\gamma =1}^{4}{\varvec{n}}^\gamma p_\gamma ^{\hbox{st}}= [0;\, 0] p_1^{\hbox{st}}+ [0;\, 1] p_2^{\hbox{st}}+ [1;\, 0] p_3^{\hbox{st}}+ [1;\, 1] p_4^{\hbox{st}}= \dfrac{1}{({E}+{H})({E}+ {I}){I}+ {E}^2{H}} \\&[({E}{I}+{H}{I}+ {E}^2){I};\, ({E}+ {I}) {H}{I}]. \end{aligned}$$

The stationary current matrix can be calculated using the state currents (8), and the found stationary probabilities (9) are

$$\begin{aligned} {\hat{\mathbf{i }}}^{\hbox{st}}= \sum _{\gamma =1}^{4}{\hat{\mathbf{i }}}^\gamma p_\gamma ^{\hbox{st}}= \dfrac{({E}+ {I}){E}{I}{H}}{({E}+ {H})({E}+ {I}){I}+ {E}^2 {H}} \begin{bmatrix} 0 &{} 1 &{} -1 &{} 0\\ -1 &{} 0 &{} 0 &{} 1\\ 1 &{} 0 &{} 0 &{} 0\\ 0 &{} -1 &{} 0 &{} 0\\ \end{bmatrix} \end{aligned}$$

Herein, Shockley–Read–Hall-like transport equations (1) describing the dynamics of average occupancy are

$$\begin{aligned} {} & \frac{{\hbox {d}\bar{n}}_1}{\hbox {d}t} = (1 - {\bar{n}}_1) {I}- {\bar{n}}_1 (1 - {\bar{n}}_2) {H};\\&\frac{{\hbox {d}\bar{n}}_2}{\hbox {d}t} = {\bar{n}}_1 (1 - {\bar{n}}_2) {H}- {\bar{n}}_2 {E}. \end{aligned}$$

The stationary state is given by the following nonlinear system of equations:

$$\begin{aligned} {} & {I}(1 - {\bar{n}}_1^{\hbox{st}}) - {H}{\bar{n}}_1^{\hbox{st}}(1 - {\bar{n}}_2^{\hbox{st}}) = 0;\\&H {\bar{n}}_1^{\hbox{st}}(1 - {\bar{n}}_2^{\hbox{st}}) - {E}{\bar{n}}_2^{\hbox{st}}= 0. \end{aligned}$$

This nonlinear system of equations can be solved analytically:

$$\begin{aligned}{} &{\bar{n}}_1^{\hbox{st}}= \dfrac{1}{2} + \dfrac{\sqrt{({E}+{H})^2{I}^2 + 2 {E}{H}({E}- {H}){I}+ {E}^2{H}^2} - ({I}+ H){E}}{2 {I}H};\\&{\bar{n}}_2^{\hbox{st}}= \dfrac{1}{2} - \dfrac{\sqrt{({E}+{H})^2{I}^2 + 2 {E}{H}({E}- {H}){I}+ {E}^2{H}^2} - ({E}+ {H}){I}}{2 {E}{H}}.\\ \end{aligned}$$

According to the SRH model, the stationary current can be found using average filling:

$$\begin{aligned} i^{\hbox{st}}= (1 - {\bar{n}}^{\hbox{st}}_1) q {I}= {\bar{n}}^{\hbox{st}}_1 (1 - {\bar{n}}^{\hbox{st}}_2) q {H}= n^{\hbox{st}}_2 q {E}= q\dfrac{{E}{I}+ {H}{I}+ {E}{H}- \sqrt{({E}+{H})^2{I}^2 + 2 {E}{H}({E}- {H}){I}+ {E}^2{H}^2}}{2 {H}}. \end{aligned}$$

### Bulk-limited current (${\bar{n}}_1 = 1$, ${\bar{n}}_N = 0$)

Consider a simple case of a uniform linear *N*-trap chain under a strong right-to-left electric field. Also consider that the current is bulk-limited when ${I}_{\mathrm{left}} \gg {H}\gg {E}_{\text{left}}$ and ${E}_{\text{right}} \gg {H}\gg {I}_{\text{right}}$, i.e. the interfaces between electrodes and a dielectric layer are transparent for charge carriers. In this case, the first trap is filled, and the last one is empty. It is easy to see that this case is identical to the case of the uniform linear chain of $N-2$ traps under a strong electric field when ${I}= {E}= {H}$ (the always filled first trap can be treated as the left electrode with ${I}= {H}$ and the always empty last trap can be treated as the right electrode with ${E}= {H}$).

#### Case of $N = 4$

This case is identical to the case of a two-trap chain (the two middle traps) with the corresponding matrices:

$$\begin{aligned} {\hat{\mathbf{P }}}= & {} \begin{bmatrix} 1 - {H}\delta t &{} 0 &{} {H}\delta t &{} 0\\ {H}\delta t &{} 1 - {H}\delta t - {H}\delta t &{} 0 &{} {H}\delta t\\ 0 &{} {H}\delta t &{} 1 - {H}\delta t &{} 0\\ 0 &{} 0 &{} {H}\delta t &{} 1 - {H}\delta t\\ \end{bmatrix}, \quad {\hat{\mathbf{Q }}} = \lim \limits _{\delta t \rightarrow 0} \dfrac{{\hat{\mathbf{P }}}^{\mathrm{T}} - {\hat{\mathbf{I }}}}{\delta t} = {H}\begin{bmatrix} - 1 &{} 1 &{} 0 &{} 0\\ 0 &{} - 2 &{} 1 &{} 0\\ 1 &{} 0 &{} - 1 &{} 1\\ 0 &{} 1 &{} 0 &{} - 1\\ \end{bmatrix},\\ {\hat{\mathbf{i }}}^1 = & {} q {H}\begin{bmatrix} 0 &{} 0 &{} -1 &{} 0\\ 0 &{} 0 &{} 0 &{} 0\\ 1 &{} 0 &{} 0 &{} 0\\ 0 &{} 0 &{} 0 &{} 0\\ \end{bmatrix}, \quad {\hat{\mathbf{i }}}^2 = q {H}\begin{bmatrix} 0 &{} 0 &{} -1 &{} 0\\ 0 &{} 0 &{} 0 &{} 1\\ 1 &{} 0 &{} 0 &{} 0\\ 0 &{} -1 &{} 0 &{} 0\\ \end{bmatrix}, \quad {\hat{\mathbf{i }}}^3 = q {H}\begin{bmatrix} 0 &{} 1 &{} 0 &{} 0\\ -1 &{} 0 &{} 0 &{} 0\\ 0 &{} 0 &{} 0 &{} 0\\ 0 &{} 0 &{} 0 &{} 0\\ \end{bmatrix},\quad {\hat{\mathbf{i }}}^4 = q {H}\begin{bmatrix} 0 &{} 0 &{} 0 &{} 0\\ 0 &{} 0 &{} 0 &{} 1\\ 0 &{} 0 &{} 0 &{} 0\\ 0 &{} - 1 &{} 0 &{} 0\\ \end{bmatrix}. \end{aligned}$$

To obtain the dynamic solution, the initial conditions should be given. Let us study the dynamic of filling the initially empty middle traps. In this case ${\varvec{p}}(0) = [1;\, 0;\, 0;\, 0]$ and equation (4) gives the following evolution of state probability values:

$$\begin{aligned} {} & p_1(t) = \dfrac{1}{5} + \dfrac{1}{2}\exp (-{H}t) + \dfrac{3\cos ({H}t) + \sin ({H}t)}{10}\exp (-2 {H}t), \qquad&(\text {probability of state } [1;\,0;\,0;\,0])\\&p_2(t) = \dfrac{1}{5} - \dfrac{\cos ({H}t) + 2 \sin ({H}t)}{5}\exp (-2 {H}t),&(\text {probability of state } [1;\,0;\,1;\,0])\\&p_3(t) = \dfrac{2}{5} - \dfrac{2\cos ({H}t) - \sin ({H}t)}{5}\exp (-2 {H}t),&(\text {probability of state } [1;\,1;\,0;\,0])\\&p_4(t) = \dfrac{1}{5} - \dfrac{1}{2}\exp (-{H}t) + \dfrac{3\cos ({H}t) + \sin ({H}t)}{10}\exp (-2 {H}t),&(\text {probability of state } [1;\,1;\,1;\,0]) \end{aligned}$$

The dynamic solution for the average trap filling can be found using the probability values:

$$\begin{aligned} \begin{aligned} {\bar{n}}_1&= 1,\\ {\bar{n}}_2&= \dfrac{3}{5} - \dfrac{1}{2}\exp (-{H}t) - \dfrac{\cos ({H}t) - 3 \sin ({H}t)}{10}\exp (-2 {H}t),\\ {\bar{n}}_3&= \dfrac{2}{5} - \dfrac{1}{2}\exp (-{H}t) + \dfrac{\cos ({H}t) - 3 \sin ({H}t)}{10}\exp (-2 {H}t),\\ {\bar{n}}_4&= 0. \end{aligned} \end{aligned}$$

The time dependence of currents between traps on time is as follows:

$$\begin{aligned} \begin{aligned} i_{12}&= \dfrac{2}{5} q {H}\left( 1 + \dfrac{5}{4}\exp (-{H}t) + \dfrac{\cos ({H}t) - 3 \sin ({H}t)}{4} \exp (-2 {H}t)\right) ,\\ i_{23}&= \dfrac{2}{5} q {H}\left( 1 - \dfrac{2 \cos ({H}t) - \sin ({H}t)}{2} \exp \{-2 {H}t\}\right) ,\\ i_{34}&= \dfrac{2}{5} q {H}\left( 1 - \dfrac{5}{4}\exp (-{H}t) + \dfrac{\cos ({H}t) - 3 \sin ({H}t)}{4} \exp (-2 {H}t)\right) . \end{aligned} \end{aligned}$$

**Figure 3:**
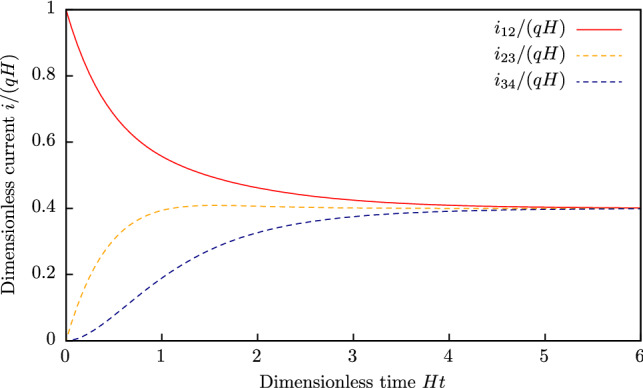
Evolution of currents between traps $i_{12}$, $i_{23}$ and $i_{34}$ for a uniform chain of four traps under strong electric field.

The evolution of currents between traps $i_{12}$, $i_{23}$ and $i_{34}$ is shown in Fig. 3. One can see that, at the beginning, the current between the left traps is quite large, while the currents between the right traps are small. In a time $t \approx 5/{H}$, a stationary state is established, and the currents become the same $i=0.4q{H}$. It should be noted that we do not take into account the quantum nature of an electron, because all processes under consideration take place at room temperature and above. Also, electron quantum states are destroyed during inelastic (de-)trapping acts and the transport is not ballistic. That is why we do not get the conductivity quantum in the obtained solutions.

It is important to note that matrices ${\hat{\mathbf{Q }}}$ and ${\hat{\mathbf{i }}}^\gamma$, in the case of the linear 4-trap chain under the strong right-to-left electric field with a bulk-limited current, are simply proportional to ${H}$. Obviously, this will be the case for the *N*-trap chain also. A couple of significant conclusions can be drawn from this fact:

- stationary average trap filling does not depend on the ${H}$ value;
- stationary current will have the form $C_i q{H}$;
- time of establishing a near-stationary solution will have the form $C_\tau /{H}$.

**Figure 4:**
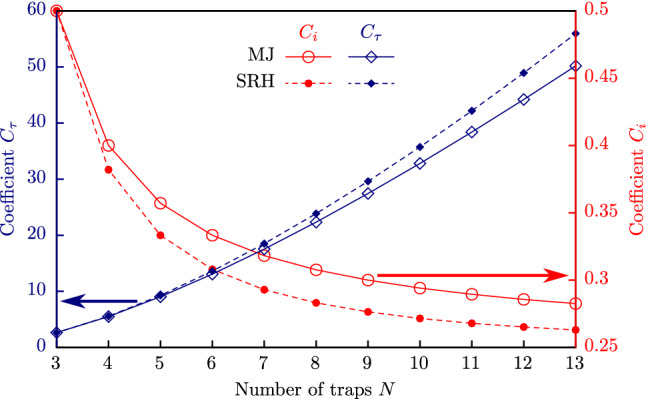
The $C_i$ and $C_\tau$ coefficients values.

Here $C_i$ and $C_\tau$ are the coefficients that depend on the trap number *N* only. The values of $C_i$ and $C_\tau$ are shown in Fig. 4. One can see that $C_i$ tends to the value 0.25 as the number of traps increases for both SRH and MJ cases, but the SRH model predicts a lower current than MJ model. Also, the Markov jump processes approach predicts longer transients (steady-state establishment) than the Shockley–Read–Hall-like approach.

**Table 1:** Comparison of analytically found stationary trap filling distributions and currents between traps for MJ and SRH models.

	Markov jump-like	Shockley–Read–Hall-like	Error (%)
${\bar{n}_{1}^{\hbox{st}}}$	1	1	0
${\bar{n}_{2}^{\hbox{st}}}$	$\dfrac{3}{5}=0.6$	$\dfrac{\sqrt{5} - 1}{2} \approx 0.618$	$\approx 3$
${\bar{n}_{3}^{\hbox{st}}}$	$\dfrac{2}{5}=0.4$	$\dfrac{3 - \sqrt{5}}{2} \approx 0.382$	$\approx -5$
${\bar{n}_{4}^{\hbox{st}}}$	0	0	0
${i}^{\hbox{st}}$	$\dfrac{2}{5}q{H}=0.4q{H}$	$q {H}\dfrac{3 - \sqrt{5}}{2} \approx 0.382q{H}$	$\approx -5$

The comparison of stationary trap fillings and the current in the case of 4-trap chain for the MJ and the SRH models is shown in Table 1. One can see that the Shockley–Read–Hall-like model gives deviations up to $5\%$ from the exact values that are calculated in terms of the finite Markov jump processes approach. On the one hand, the obtained deviations of the current might be smaller than different noises in real experiments, but they might be too large for simulations of high-quality electronic devices.

**Figure 5:**
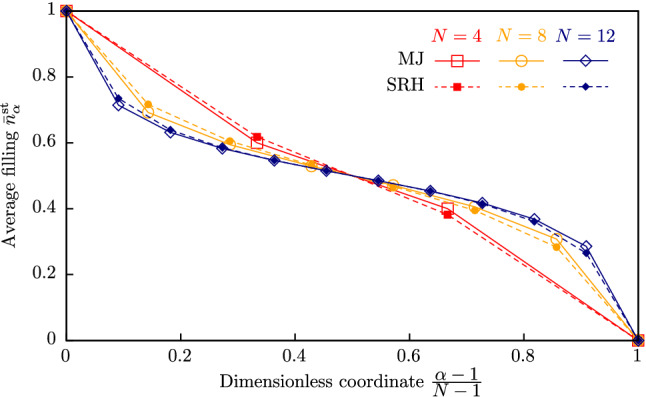
Trap filling distributions, calculated in terms of SRH and MJ approaches for $N=4$, 8 and 12.

The same procedure can be carried out for larger trap chains. The comparison of stationary trap filling distributions calculated within the SRH and the MJ models for trap chains with $N=4$, 8 and 12 is shown in Fig. 5. One can see that the shape of all curves is close and it tends, with the increase in *N*, to that shown in Figure 3 in^25^ for the continuum case.

## Discussion

We have developed a discrete model of the charge transport in dielectrics in terms of finite Markov jump processes, and it takes into account hops of localized charge carriers between traps in a dielectric medium in strong electric fields with the injection from and extraction to electrodes (contacts). The developed model is formulated as a matrix differential equation or a system of linear algebraic equations. The dynamic solutions describing the charge transport are found using a matrix exponential. The stationary solutions describing the charge transport are found by solving a system of linear equations. The major drawback of the presented method for the charge current description is the dramatic rise in the computational complexity with the increasing amount of trap. However, an electronic system, containing a dozen of traps, can be easily simulated using a laptop PC. Moreover, we demonstrated that the solutions can be acquired by manipulations with sparse matrices. This means that the numeric computation of transition processes can be solved quite quickly using optimized algorithms^26,27^. The developed model is universal and can be applied to a relatively large number of traps distributed in 1D, 2D or 3D systems. Also, the matrix sparsity can be increased by ignoring extremely long hops in a specific spatial trap distribution, thus, reducing the total difficulty of the task.

The analytical stationary solutions, that are close to experimentally achievable conditions, are found. The comparison with other theoretical models^25^ shows a good agreement, which proves their good applicability, at least, for the stationary problems under examination. Also, the presented model allows one to get exact analytical solutions of the charge transport evolution in time, while Shockley–Read–Hall-like transport equations require their integration to get the same results. This procedure is more difficult: it requires more computational resources and time. So, the advantage of the presented solutions over others lies in the fact that it can be used as an exact reference to test other (approximate) solutions in both dynamic and stationary cases.

The developed model, formulated in linear algebraic equations, can be implemented in the computational code using different libraries, such as BLAS, LAPACK, MKL, SciPy/NumPy etc. Particularly, the proposed approach can enhance the current implementation of the leakage current simulations through dielectric layers in the Synopsys TCAD software package^28^. It was shown that the stationary current-voltage characteristics of metal-insulator-semiconductor and metal-insulator-metal structures can be calculated analytically for any trap distribution. Also, the model implemented as a numeric code can be used in simulations of flash, FRAM, RRAM memories and memristor resistive switching with different spatial designs, including vertical and planar ones.
